# The Role of T Cells and Macrophages in Asthma Pathogenesis: A New Perspective on Mutual Crosstalk

**DOI:** 10.1155/2020/7835284

**Published:** 2020-08-19

**Authors:** Xueyi Zhu, Jie Cui, La Yi, Jingjing Qin, Wuniqiemu Tulake, Fangzhou Teng, Weifeng Tang, Ying Wei, Jingcheng Dong

**Affiliations:** ^1^Department of Integrative Medicine, Huashan Hospital, Fudan University, Shanghai 200040, China; ^2^Institutes of Integrative Medicine, Fudan University, Shanghai 200040, China

## Abstract

Asthma is associated with innate and adaptive immunity mediated by immune cells. T cell or macrophage dysfunction plays a particularly significant role in asthma pathogenesis. Furthermore, crosstalk between them continuously transmits proinflammatory or anti-inflammatory signals, causing the immune cell activation or repression in the immune response. Consequently, the imbalanced immune microenvironment is the major cause of the exacerbation of asthma. Here, we discuss the role of T cells, macrophages, and their interactions in asthma pathogenesis.

## 1. Introduction

Asthma is a common chronic airway disease characterized by airway inflammation, hyperresponsiveness, and variable airway obstruction, which is often attributed to gene-environment interactions [1, 2]. There are approximately 334 million people worldwide suffering from asthma [3]. Multiple immune cells are involved with the development of asthma, such as T cells, macrophages, dendritic cells, eosinophils, neutrophils, mast cells, and basophils [4]. Eosinophilic asthma, neutrophilic asthma, and mixed granulocytic asthma are all influenced by cytokines and chemokines from these immune cells [5]. Recent studies have highlighted that the imbalance of T cells or macrophage dysfunction contributes to the progression of asthma [6]. However, uncertainty remains as to the crosstalk of these two cells. To shed light on this perspective, we summarize the role of both T cells and macrophages as well as their interactions in asthma pathogenesis, hoping to provide a basis for potential targets in the future treatment of asthma. We also speculate that extracellular vesicles might be the main mediator of their crosstalk. Alternatively, the corresponding cytokine storm is probably involved with severe asthma.

## 2. Imbalance of T Cells in Asthma Pathogenesis

**Figure 1:**
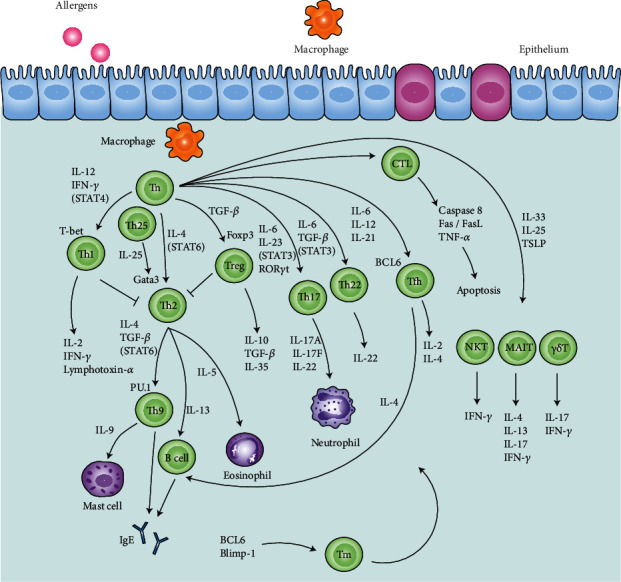
Imbalance of T cells in asthma pathogenesis. In response to allergens, the naïve T (Tn) cells are activated by the macrophages and tend to differentiate into T helper (Th) 1, Th2, Th17, Th22, Th9, Th25, T regulatory (Treg), T follicular helper (Tfh), natural killer T (NKT), mucosal-associated invariant T (MAIT), *γδ*T cells, cytotoxic CD8^+^T lymphocytes (CTLs), and memory T (Tm) cells. They secrete cytokines to activate and recruit the eosinophils, neutrophils, mast cells, and B cells. The arrows represent the secretions from these cells, affecting the progression of asthma. The transcriptional factors T-bet, Gata3, RAR-related orphan receptor (ROR) *γ*t, Foxp3, and PU.1 are necessary to Th1, Th2, Th17, Treg, Th22, and Th9 cell activation. The signal transducer and activator of transcription (STAT) signals are involved in the process. Besides, caspase 8 and the Fas/FasL pathway are related to cell apoptosis mediated by CTLs.

T cells, derived from lymphoid stem cells in the bone marrow, participate in antigen-specific responses. When naïve T (Tn) cells encounter the antigen, they have the potential to differentiate into effector T cells and a small portion of memory T cells. Effector T cells include T helper (Th) 1, Th2, Th17, Th22, Th9, Th25, T regulatory (Treg), T follicular helper, natural killer T cells, and cytotoxic CD8^+^T lymphocytes [7]. They not only regulate innate immune cells (macrophages, eosinophils, mast cells, basophils, etc.) but also stimulate B cells to resist viruses. T cells also generate enormous cytokines and chemokines to amplify the immune response [8, 9], thus enhancing airway smooth muscle contraction, mucus secretion, and airway hyperresponsiveness (AHR), as well as T cell proliferation in asthma [10]. Here, we review the detailed role of these cells in asthma (Figure 1).

### 2.1. Th Cells and Treg Cells

#### 2.1.1. Th1/Th2 Imbalance in Eosinophilic Asthma

Th1/Th2 imbalance plays a significant role in asthma pathogenesis. Interleukin- (IL-) 12 and interferon- (IFN-) *γ* induce T-bet to stimulate Th1 cells through the signal transducer and activator of transcription (STAT) 4 signal while IL-4 induces Gata3 to activate Th2 cells via the STAT6 signal [11]. Th1 cells generate IL-2, IFN-*γ*, and lymphotoxin- (LT-) *α*, promoting type 1 immunity. They have dual regulatory roles in asthma: they repress Th2 cell activation to confine eosinophilic inflammation but advance neutrophilic inflammation [12]. Raundhal et al. [13] revealed that IFN-*γ*^+^Th1 cells were dominant in severe asthma, potentially associated with corticosteroid unresponsiveness. Th1 cell recruitment to the inflammatory sites can be enhanced by CXC chemokine ligand (CXCL) 10 and its cognate CXC chemokine receptor (CXCR) 3, leading to corticosteroid resistance [14]. Notably, Th2 cells are directly triggered by airway epithelial-derived cytokines (IL-33, IL-25, and thymic stromal lymphopoietin (TSLP)) [15], subsequently secreting Th2-associated cytokines (IL-4, IL-5, and IL-13) [16, 17]. IL-4 and IL-13 urge B cells to produce IgE, mucus secretion, and AHR [18, 19]. IL-5 maintains the survival and vitality of eosinophils, dominant in eosinophilic inflammation and AHR [20]. Moreover, Th2-associated chemokine receptors (CCR3, CCR4, and CCR8) and ligands (CCL1, CCL5, CCL26, and CX3CL1) assist in eosinophil recruitment and AHR [21, 22]. CCL17 and CCL22 also have the capability to increase Th2 cell proliferation via autocrine loops [23]. Although corticosteroid therapies have a great effect on alleviating Th1/Th2 imbalance in asthma, more attention has currently been paid to a range of treatments targeting Th2-associated cytokines, such as mepolizumab, reslizumab, and lebrikizumab [24, 25].

#### 2.1.2. Th17/Treg Imbalance in Neutrophilic Asthma

Recent studies have demonstrated that Th17/Treg imbalance also remains vital in asthma pathogenesis. IL-6 and IL-23 induce RAR-related orphan receptor (ROR) *γ*t to activate Th17 cells through the STAT3 signal whereas transforming growth factor- (TGF-) *β* expresses Foxp3 to promote Treg cell differentiation [26]. Alcorn et al. [27] discussed that Th17 cells produced IL-17A, IL-17F, and IL-22 via the toll-like receptor (TLR) 4/IFN-*β* (TRIF) pathway, exerting proinflammatory function in neutrophil recruitment and activation. Interestingly, IL-17 has dual regulatory abilities: it recruits neutrophils to the inflammatory site to protect the lungs but aggravates neutrophilic asthma [28, 29]. Rahman et al. [30] found that IL-17A modulated the rapid phosphorylation of mitogen-activated protein kinase (MAPK), expressing eotaxin-1/CCL11. Moreover, the bond between CCR4 or CCR6 and CCL20 enhances Th17 cell recruitment to the lesion [31]. CD25^+^CD4^+^Foxp3^+^Treg cells, classified into thymus-derived natural Treg (nTreg) and peripheral induced Treg (iTreg) cells, secrete anti-inflammatory factors IL-10 and TGF-*β*. They regulate the immune response to avoid overactivation, promote immune tolerance, and maintain balance in the immune microenvironment [32]. Associated chemokine receptors (CCR4, CCR5, CCR7, and CCR8) enhance Treg cell recruitment [33]. There is also a large amount of IL-35-induced Treg cells, called iTr35 cells. They serve as immunomodulators to suppress the inflammatory response [34]. The Th17/Treg imbalance mechanism, which leads to neutrophilic, severe, or corticosteroid-resistant asthma, is a supplement to the Th1/Th2 imbalance mechanism in asthma [35, 36]. Of note, novel therapeutics targeting these cytokines have gained more and more attention in corticosteroid-insensitive asthma [37].

#### 2.1.3. Th22 Cells

Th22 cell differentiation, closely associated with Th17 cells, is triggered by IL-6 and TGF-*β* via STAT3 signaling [38]. Besnard et al. [39] proved that the level of IL-4, IL-5, IL-13, and IL-33; counts of eosinophils and neutrophils; and AHR were downregulated in ovalbumin- (OVA-) induced IL-22^−^ mice, which implied that IL-22 had a proinflammatory ability. However, IL-22 also has a protective role in the absence of IL-17, which is probably correlated with the production of IL-10 [40]. CCR4 and CCR6 further assist the secretion of IL-22 from Th22 cells to enhance epithelial proliferation and repair the barrier function of the mucosal surface via nuclear factor kappa-B (NF-*κ*B) signaling [41].

#### 2.1.4. Th9 or Th25 Cells

Th2 cells express the transcriptional factors PU.1 and interferon regulatory factor (IRF) 4 in the presence of IL-4 and TGF-*β*, thus driving Th9 cell activation via the STAT6 signal [42–44]. Reversely, Th9 cells are suppressed by the overexpression of B lymphocyte-induced maturation protein 1 (Blimp-1) [45]. Th9 cells produce IL-9 to recruit and activate mast cells and B cells, associated with IgE elevation and corticosteroid resistance [46, 47]. They also interact with innate lymphoid cell (ILC) 2 to further amplify type 2 inflammation. IL-25 (IL-17E), originated from Th25 cells, also has a close relationship with Th2 cells: it can drive Th2 cell differentiation [48].

#### 2.1.5. T Follicular Helper Cells

IL-6, IL-12, and IL-21 elevate T follicular helper (Tfh) cell differentiation with the combination of inducible T cell costimulator (ICOS) and OX40, expressing B cell lymphoma (BCL) 6 [49]. CD4^+^CXCR5^+^Tfh cells secrete IL-4 to drive B cells and the subsequent production of immunoglobulin (IgE) [50]. Also, IL-2, IL-4, and IL-13 secreted by Tfh cells expand type 2 inflammation [51, 52].

### 2.2. Cytotoxic CD8^+^T Lymphocytes

Cytotoxic CD8^+^T lymphocytes (CTLs) can be classified into the subtypes similar to the subtypes of Th cells and corresponding functions. As we all know, the CD4/CD8 ratio is considerably higher in asthma; how to maintain it balanced is a key to therapy. CTLs search for and recognize specific antigens presented by major histocompatibility complex (MHC) class I molecules and then combine them with T cell receptor (TCR), thus forming complexes. Next, chemicals such as perforin and granzyme are released through direct contact while IgE is correspondingly suppressed. Chávez-Galán et al. [53] pinpointed that CTLs mediated cell apoptosis via caspase 8 and the Fas/FasL pathway, which is also related to the effect of tumor necrosis factor- (TNF-) *α*. Together with all the influence above, type 1 inflammation is amplified while type 2 inflammation is weakened by CTLs [54].

### 2.3. Innate-Like T Cells

Innate-like T (ILT) cells, including natural killer T (NKT), mucosal-associated invariant T (MAIT), and *γδ*T cells, belong to unconventional T cell subgroups [55]. They all have similar functions and express *αβ* or *γδ* TCR chains, which can be activated in a TCR-dependent or TCR-independent manner [56]. They generate IL-4, IL-5, IL-13, and IFN-*γ* to develop Th2 and Th17 cell activations, modulating eosinophilic infiltration and AHR [57]. Meanwhile, IL-17 is secreted to modulate neutrophilic asthma and macrophage proliferation. Lezmi and Leite-de-Moraes [58] confirmed that NKT cells also regulated CTLs and killed infected cells as analogous to CTLs in asthmatic patients.

### 2.4. Memory T Cells

Memory T (Tm) cells develop immune memory and train the body to react rapidly during the secondary immune response. BCL6, Blimp-1, and histone H3 lysine 4 (H3K4me2) participate in Tm cell differentiation [59, 60]. It was demonstrated that IL-2-resident CD4^+^ Th2 memory cells promoted eosinophilic asthma and produced IL-5 via IL-33-ST2-p38 kinase signaling [61–63]. Tm cells express IL-17 and CCR7 as well [64]. Furthermore, Tm cell-induced inflammatory response is amplified via IL-25 but suppressed through IL-35 [65].

### 2.5. Other T Cells (Intraepithelial Lymphocytes and Jurkat T Cells)

It was described that CD103^+^CD69^+^ intraepithelial lymphocytes (IELs) were remarkably activated in the sputum of asthmatic patients [66]. CD4^+^CD103^+^ IELs always remain in the inflammatory tissues. On the other side, Jurkat T cells are one of the T cell lineages, often used for functional verifications in the studies of asthma [67].

## 3. Macrophage Dysfunction in Asthma Pathogenesis

**Figure 2:**
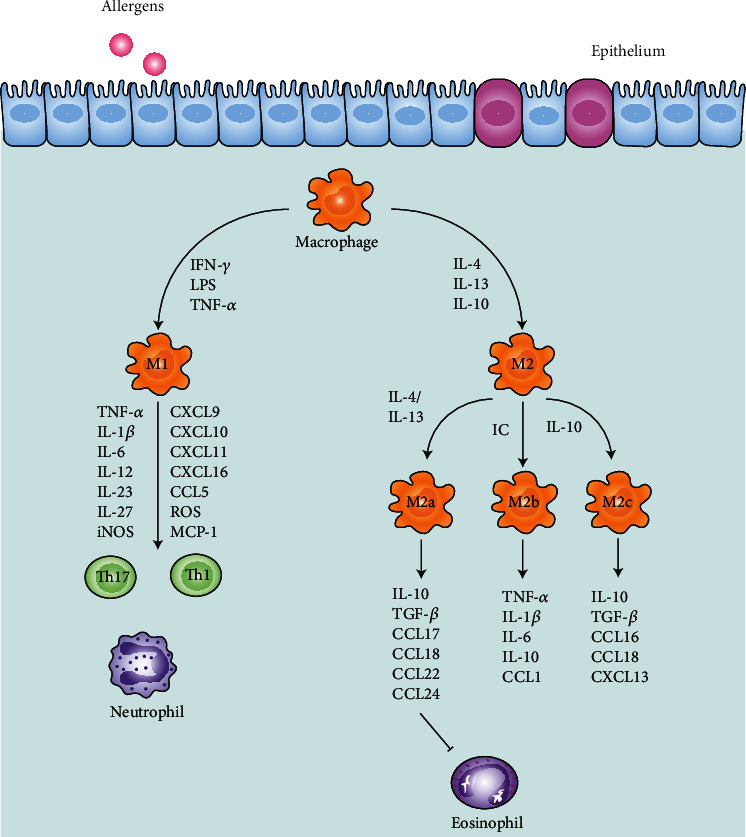
Macrophage dysfunction in asthma pathogenesis. In response to allergens, macrophages differentiate into classically activated (M1) and alternatively activated (M2) macrophages. The arrows refer to the cytokines and chemokines secreted by macrophages, including tumor necrosis factor- (TNF-) *α*, interleukin (IL), and interferon- (IFN-) *γ*. M1 macrophages produce T helper (Th) 1-associated and Th17-associated cytokines, affecting the neutrophils. M2 macrophages further differentiate into M2a, M2b, and M2c macrophages, affecting the eosinophils.

Macrophages are primarily derived from monocytes in the bone marrow and present in almost all tissues, mainly classified into alveolar macrophages (AMs) and interstitial macrophages (IMs) in the lungs [68]. They not only phagocytose and directly kill antigens to mediate innate immunity but also process and present antigens to assist adaptive immunity in asthma [69]. They produce the oxygen radicals to decrease *β*-adrenergic response and thromboxane A2 to enhance cholinergic response. It is like a double-edged sword in the inflammation of asthma. Lee et al. [70] demonstrated that the lack of AMs led to the suppression of type 2 inflammation and airway remodeling. However, it was argued that the adoptive immunity of AMs to asthmatic mice attenuated airway hyperreactivity in asthmatic mice. Other mice with removed AMs had worsening lung function and Th2-type inflammatory response exacerbation [71]. It has been generally accepted that macrophage polarization breaks the balance of classically activated (M1)/alternatively activated (M2) macrophages in asthma [72, 73]. IFN-*γ*, lipopolysaccharide (LPS), and TNF-*α* stimulate M1 macrophages while IL-4, IL-13, and IL-10 activate M2 macrophages [74]. CCL2 and CXCL4 help develop M2 polarization as well [75] (Figure 2).

### 3.1. M1 Polarization and Proinflammation in Asthma Pathogenesis

M1 macrophages highly express MHC class II molecules, CD80, CD86, TLR4, and inducible nitric oxide synthase (iNOS), further producing Th1-associated cytokines (TNF-*α*, IL-1*β*, IL-2, IL-6, and IL-12), Th17-associated cytokines (IL-23 and IL-27), monocyte chemotactic protein- (MCP-) 1, reactive oxygen species (ROS), and chemokines (CXCL9, CXCL10, CXCL11, CXCL16, CCL2, CCL5, and CCL8). M1 macrophages exacerbate the Th1-type and Th17-type inflammatory response, linked with neutrophilic infiltration, corticosteroid resistance, oxidative damage, and AHR [76, 77]. Goleva et al. [78] gave the proof that M1 macrophages had a higher expression in bronchoalveolar lavage fluid (BALF) of corticosteroid-resistant asthmatic patients. Interestingly, the macrophages are characterized by heterogeneity and plasticity that M1 macrophages can differentiate into M2 macrophages.

### 3.2. M2 Polarization and Its Modulation of Inflammation in Asthma Pathogenesis (a Double-Edged Sword)

M2 macrophages have the lower expression of MHC class II molecules and CD86 as well as the higher expression of macrophage mannose receptor (MRC) 1, arginase (Arg) 1, CD206, and CD163, modulating eosinophilic infiltration in type 2 inflammation [79]. They are prominent in parasite immunomodulation, tissue remodeling, and Th2 cell differentiation [80]. Melgert et al. [81] observed that M2 macrophages were abundant in the BALF of asthmatic patients. Furthermore, they demonstrated that allergen-induced disease was exacerbated after the adoptive transfer of M2 macrophages [82]. NOD-, LRR-, and pyrin domain-containing (NLRP) 3 inflammasome was proved to have the potential to upregulate IL-4 to promote M2 polarization [83]. Specifically, IL-4 or IL-13 induces M2a macrophages to express IL-10, TGF-*β*, and chemokines (CCL17, CCL18, CCL22, and CCL24), which participate in Th2-type inflammation, airway remodeling, and parasite immunomodulation. Immune complex (IC) promotes M2b macrophage activation, taking part in Th2-type immune regulation and expressing TNF-*α*, IL-1*β*, IL-6, IL-10, and CCL1. IL-10 or prostaglandin (PG) E2 stimulates monocytes to differentiate into M2c macrophages; they further express IL-10, TGF-*β*, CCL16, CCL18, and CXCL13, repressing inflammation and enhancing tissue repair [84].

### 3.3. Innate Immune Response Mediated by Macrophages

Macrophages act as the first-line defense in the lungs and the initial responders to immune stimulation, belonging to the innate immune system [85]. Pattern recognition receptors (PRRs) on macrophages, particularly TLRs, recognize pathogen-associated molecular patterns (PAMPs) or damage-associated molecular patterns (DAMPs) on antigens. Then, antigens are endocytosed in macrophages through the interactions and lysosomes are released to kill them. After that, the protein residues are presented on the macrophage surface, forming MHC molecules. It has been pointed out that macrophages regulate the inflammatory microenvironment via autocrine or paracrine [86]. They have the ability to secrete PGE2, PGD2, and D-prostanoid receptor (DP) 1 to enlarge neutrophil migration [87, 88]. Alternatively, macrophage apoptosis, autophagy, and necrosis are closely related to immune response exacerbation or alleviation, probably caused by mitochondrial autophagy or endoplasmic reticulum (ER) stress [89].

## 4. Crosstalk between T Cells and Macrophages in Asthma Pathogenesis

### 4.1. Adaptive Immune Response Mediated by T Cells and Macrophages (Cellular Immunity)

**Figure 3:**
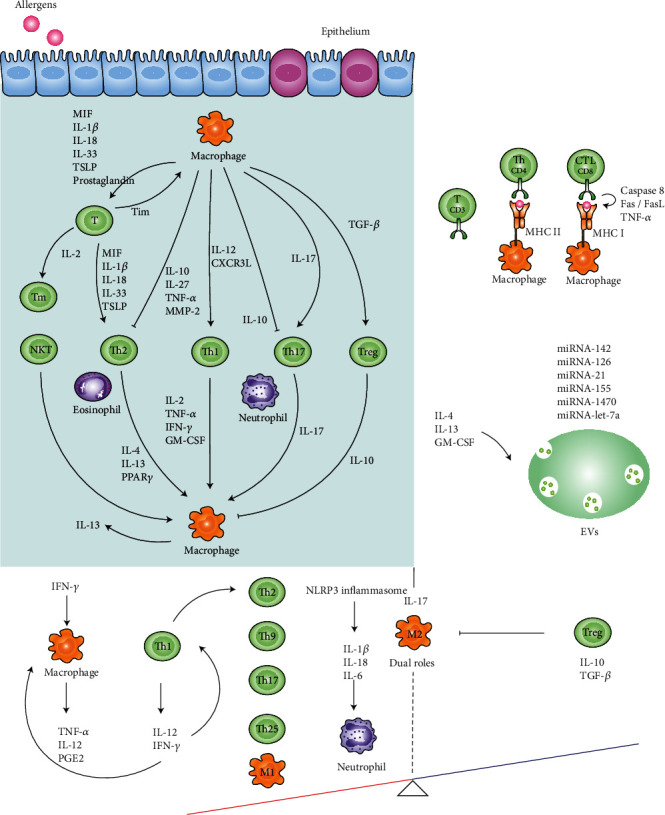
Crosstalk between T cells and macrophages in asthma pathogenesis. The arrows represent the cytokines secreted by T cells and macrophages. Macrophages secrete interleukin- (IL-) 10 to repress T helper (Th) 2 and Th17 cells. Similarly, Treg cells secrete IL-10 to inhibit macrophages. Antigen-contained major histocompatibility complex (MHC) class I or II molecules on macrophages bind to the T cell receptor (TCR) on T cells to mediate the adaptive immune response. Extracellular vesicles (EVs) carrying miRNAs (miRNA-142, miRNA-126, miRNA-21, etc.) may be the modulators of the crosstalk between T cells and macrophages. In addition, the imbalance caused by the cytokine storm may be the main cause of severe asthma. The red side refers to the positive feedback loops while the blue side refers to the negative feedback loops.

If the antigens cannot be eliminated in the innate immune response, they will be processed to constitute MHC class I or II molecules and presented on the macrophage surface [90, 91]. Then, the TCR of T cells recognizes and connects them, further jointing with costimulatory molecules. A large number of cytokines are gathered to switch on the adaptive immune response. Macrophage migration inhibitory factor (MIF) participates in the modulation of both the innate and adaptive immune responses [92]. Immunological synapse includes B7-1/2 (CD80/CD86) on macrophages, which binds to CD28 and cytotoxic T lymphocyte-associated protein- (CTLA-) 4 on Tn cells [93]. ICOS matches ICOS-L on Tm cells while OX40 contacts to OX40L [94]. In this process, T cell proliferation is further expanded, and macrophages are also affected (Figure 3).

#### 4.1.1. Macrophages Activate and Suppress T Cell Functions

The antigens on MHC molecules from macrophages ensure CD4^+^T cell activation and proliferation in an antigen-specific manner [95]. Bozza et al. [96] proved that macrophages secreted MIF and the lack of MIF reduced activated antigen-specific CD4^+^T cells and Th2-associated cytokines, which implied that macrophages might have functions of initiating T cell-mediated immune response. Macrophages also produce prostaglandin and interleukin in an autocrine manner and act on T cells in a paracrine manner [97]. Specifically, macrophages modulate type 1 inflammation. It was confirmed that Th1 cells could be gathered through IFN-*γ*-dependent CXCR3 ligands from macrophages [98]. AMs secrete IL-12 to amplify type 1 inflammation [99]. Conversely, M2 macrophages function to suppress type 1 inflammation [100]. Turning to Th2 cells, macrophage-derived cytokines (IL-1*β* and IL-6) and chemokines (CCL1 and CCL22) were identified to promote Th2 cell differentiation from CD4^+^T cells and recruit Th2 cells, mediating eosinophilic inflammation with the increase of IL-4 and IL-13 [101]. Lai et al. [102] indicated that IMs expressed TSLP, which was the stimulus of Th2 cell differentiation. IL-18 from macrophages was described to drive type 2 inflammation, Th2 cell differentiation, and AHR [103]. Wills-Karp et al. [104] also observed that IL-33 from macrophages drove Th2-type inflammation, urged by trefoil factor 2 (TFF2) in the house dust mite-induced asthmatic murine model. Additionally, Laidlaw and Boyce [105] proposed that macrophages synthesized and released the leukotriene (LT) C_4_, which induced Th2 cells and modulated Th2-type inflammation. Takahashi et al. [106] verified that matrix metalloproteinase- (MMP-) 2 had the protective function in allergic asthma, probably through its regulation of M1 macrophage differentiation and further downregulation of Th2-type inflammation. It can be concluded that macrophages can affect both type 1 and type 2 inflammation. Kim et al. [107] confirmed that TNF-*α*, mainly produced by activated macrophages, alleviated Th1-associated and Th2-associated cytokines, IgE, and airway remodeling. On the other side, macrophages are involved in Th17-associated immune response. Song et al. [108] demonstrated that AMs of asthmatic patients increased IL-17, which means there was also an exact relationship between macrophages and Th17 cells, especially in neutrophilic inflammation. Macrophage-associated CCL2, CCL17, and CCL22 promote T cell migration and the progression of inflammation [109]. Besides these promotions of the inflammation, a portion of macrophages has anti-inflammatory functions and modulates the excessive immune response. Kawano et al. [110] illustrated that IL-10 of IMs reduced Th2 and Th17 cells to alleviate neutrophilic asthma via the TLR4/MyD88 pathway. Chakarov et al. [111] also proposed that IMs enhanced T cell proliferation and Treg cell differentiation. In the presence of retinoic acid, AM-associated TGF-*β* develops Treg cell differentiation [112]. Lu et al. [113] explained that the anti-inflammatory function of IL-27 from M1 macrophages relied on its suppression of Th2-type inflammation via the STAT1 and STAT3 pathways.

#### 4.1.2. T Cells Activate and Suppress Macrophage Functions

Similar to macrophage controlling T cells mentioned above, T cells influence macrophage production, activation, and function. M1 macrophage differentiation is mainly promoted by Th1-associated cytokines (IFN-*γ* and LPS), and M2 macrophage differentiation is dependent on Th2-associated cytokines (IL-4 and IL-13). Kuchroo et al. [114] proposed that the regulation of macrophage vitality relied on the T cell immunoglobulin and mucin domain- (Tim-) 1 and Tim-3 on T cells. Th cell-secreted granulocyte-monocyte colony-stimulating factor (GM-CSF) initiates macrophage activation and secretion [115]. Th1-derived IFN-*γ*, IL-2, and TNF-*α* function together to enhance macrophage endocytosis function, bactericidal capacity, and proliferation [116]. Mukhopadhyay et al. [117] indicated that TNF-*α* from T cells elevated intercellular cell adhesion molecule-1 (ICAM-1), which ensured that macrophages passed through the blood vessel wall to the inflammatory site. Also, Th2-associated IL-4 motivates macrophage endocytosis ability [118]. Edukulla et al. [119] proved that Th2-associated IL-4 and IL-13 upregulated IL-31 receptor *α* expression of macrophages, enhancing M2 macrophages. Nobs et al. [120] demonstrated that Th2 cells predominantly expressed the transcription factor peroxisome proliferator-activated receptor (PPAR) *γ*, increasing M2 macrophages and AMs, which might be closely associated with IL-33-driven type 2 inflammation. Banerjee and Henderson [121] verified that in the response to the stimulus, the NADPH oxidase catalytic subunit Nox2 (gp91^phox^) alone or gp91^phox^ and MMP-12 together controlled T cells and regulated Th2-type immunity, then mediating macrophages. It can be seen that gp91^phox^ and MMP-12 may be the targets of restricting the crosstalk between T cells and macrophages. On the aspect of Th17-type immune response, IL-17 stimulates macrophages to amplify antibacterial capability through IL-17RA. IL-17A induces the production of IL-33, and their cooperation increases CXCR2-expressed macrophages, aggravating neutrophilic asthma [122]. Activated NKT cells directly urge macrophage to secrete IL-13 via the contact of invariant TCR-CD1d [123]. They also elevated the IL-33 of AMs via the IL-33/ST2 signaling pathway, mediating AHR [124]. Conversely, macrophage proliferation can be suppressed by IL-10 and TGF-*β* of Treg cells [125] (Table 1).

### 4.2. Humoral Immunity Mediated by B Cells, T Cells, and Macrophages

Macrophages and T cells assist B cell-modulated humoral immunity. After the antigens directly bind to MHC class II molecules on the B cells, the membrane-bound antibodies are presented on the B cell surface, setting the markers for macrophages to recognize and further stimulate B cell proliferation [126]. Meanwhile, receptors on the T cells recognize these presented antigens and promote B cell activation [127]. Next, a part of B cells differentiates into plasma cells and expresses antibodies via rough ER while a small proportion of them turns into memory B cells, ensuring secondary immune response. In normal circumstances, all the immune cells keep the balance and the immune microenvironment is in a stable state.

### 4.3. Immune Evasion and Cytokine Storm in Severe Asthma

If macrophages miss recognizing antigens, this might cause immune evasion [128]. On the contrary, we assume that asthma exacerbation might be associated with the cytokine storm like other infectious diseases [129]. First, antigens promote IFN-*γ* to activate macrophages and then produce TNF-*α*, IL-12, and PGE2, constituting a positive feedback loop [130]. Referring to another positive feedback loop, NLRP3 inflammasome is switched on to generate IL-1*β* and IL-18, recruiting neutrophils to the inflammatory site [131]. Kim et al. [132] described that the elevation of IL-1*β* and IL-18 via NLRP3 inflammasome led to corticosteroid-resistant asthma and is possibly involved with IL-6 [133]. Meanwhile, in the adaptive immune response, Th1 cells release IL-12 and IFN-*γ*. They stimulate Th1 cell differentiation and macrophage activation, forming another positive feedback loop to amplify defense ability [134]. Once antigens are controlled, the response is correspondingly weakened by the transmitted signals, forming a negative feedback loop. IL-10 and TGF-*β* also have the immunosuppressive effect, forming another negative feedback loop [135]. Nevertheless, if the positive feedback loops far exceed the negative feedback loops or the negative ones are too weak to maintain the balance, the immune system will release a mass of cytokines with the wrong signals, causing asthma to worsen [136, 137].

### 4.4. Role of Extracellular Vesicles in the Crosstalk between T Cells and Macrophages

Extracellular vesicles (EVs), divided into exosomes, microvesicles, and apoptotic bodies, can directly shuttle back and forth freely between the cells, which have the potential to be the mediators of crosstalk between the immune cells [138, 139]. Through EVs, the immune cells interact with each other in an autocrine, paracrine, or remote regulatory manner in asthma progression [140, 141]. It was proved that exosomes carried the nucleic acids (mRNAs and miRNAs) to achieve material delivery [142, 143]. Their characteristics enable them to be accessible to drug delivery in asthma [144]. Hence, we focus on the functions of EVs in the regulation of T cells and macrophages in asthma pathogenesis.

Some researchers have explored the relationship between EVs and macrophages in asthma pathogenesis. EVs in the lung are primarily derived from epithelial cells and macrophages [145], which can be detected in the sputum and BALF of asthmatic patients [146]. Mohan et al. [147] discovered that exosomal miRNAs (miRNA-let-7a, miRNA-21, miRNA-658, miRNA-24, miRNA-26a, miRNA-99a, miRNA-200c, and miRNA-1268) lay a great difference between asthmatic patients and healthy controls, possibly related to the severity of the allergy and the lung function. Several EVs are able to control macrophage differentiation. Ismail et al. [148] found that macrophage-secreted microvesicles contained miRNA-223, which modulated macrophage differentiation. Kulshreshtha et al. [149] observed that macrophage differentiation and proliferation were induced by the increased production of exosomes with the influence of IL-13. GM-CSF-stimulated microvesicles were also proved to motivate macrophage differentiation. Macrophage-produced exosomes have abundant functions in the immune response. It was demonstrated that IL-4 and IL-13 promoted miRNA-142 in exosomes from macrophages, which were essential to T cell development [150]. These exosomes repressed IFN-*γ* and developed memory CD4^+^T and CD8^+^T cells. Esser et al. [151] described that exosomes from macrophages contained leukotriene synthesis enzymes that were conducive to leukotriene biosynthesis, granulocyte recruitment, and proinflammation in asthma. Additionally, Paredes et al. [152] confirmed that BALF exosomes from asthmatic patients enhanced IL-8 and LTC_4_, amplifying the production of leukotriene. In the field of their anti-inflammation functions, AM exosomes were pointed out to be elevated by miRNA-133a-3p or miRNA-126-3p, delivering suppressor of cytokine signaling 3 (SOCS3) to decrease Th2-associated cytokines (IL-4 and IL-13) and alleviate allergic airway inflammation [153]. miRNA-29c from macrophages was proposed to inhibit Th2/Th17 cell differentiation via the miRNA-29c/B7-H3 axis; its feature of acting as exosome needs further exploration [154].

Besides, most studies have discussed more on the correlation between EVs and T cells in asthma pathogenesis. On the proinflammatory side, exosomes isolated from asthmatic patients expressed MHC I and MHC II molecules, costimulatory molecules (CD86), and tetraspanin proteins (CD81 and CD19), inducing T cell proliferation and releasing Th2-associated cytokines (IL-5 and IL-13) [155]. miRNA-126 was found to be higher in peripheral blood exosomes from asthmatic patients, correlated with the elevation of IL-4 and IgE [156]. It was indicated that miRNA-21 was lower in exosomes isolated from the exhaled breath condensate of asthmatic patients that increased Th2 cells and diminished Th1 cells [157]. Also, a higher expression of exosomal miRNA-155 in mild asthmatic patients seems to break the equilibrium of Th1/Th2 [158, 159]. However, it was argued that miRNA-155 in exosomes had the anti-inflammatory function that suppressed IL-13 and IL-4 to attenuate type 2 inflammation [160]. On the anti-inflammatory side, Kumar et al. [161] indicated that miRNA-let-7 repressed IL-13 and inhibited type 2 inflammation. Chiou et al. [162] demonstrated that after T cell activation, the EVs isolated from T cells contained tRNA fragments (tRFs), probably removing immune activation. Notably, the mesenchymal stem cell (MSC) generates exosomal miRNA-1470, which remains important in CD4^+^CD25^+^Foxp3^+^Treg differentiation [163]. Du et al. [164] proved that exosomes released from the MSC increased IL-10 and TGF-*β*1, thus helping asthmatic patients restore Treg cell proliferation and immunosuppressive functions. Collectively, the above findings emphasize the potential of EVs to mediate immune cell crosstalk in asthma.

## 5. Conclusion

The imbalance of the immune microenvironment is the main cause of asthma pathogenesis, in which T cells and macrophages engage in from the preliminary stage to the final curtain. The role of T cells, macrophages, and their crosstalk is of great importance in asthma. EVs show great potential as the mediators of this crosstalk. We have proposed a theoretical basis on the hypothesis of the connection between the cytokine storm and the severe asthma; further effort is required to explore this aspect. Based on the current treatment of asthma from corticosteroid to antibody therapy, figuring out the role and the crosstalk between these two cells contribute to developing the novel treatments of asthma. Targets on the T cell-associated and macrophage-associated cytokines or chemokines or miRNAs are critical to attenuating asthma and achieving personalized therapy.

## Figures and Tables

**Table 1:** Cytokines participating in the crosstalk between T cells and macrophages.

Cytokines	Derivation	Interactions between T cells and macrophages	References
IL-1*β*	Macrophages	Promote CD4^+^T cells, differentiate, and recruit Th2 cells	[101]..................
IL-2	Th1 cells	Enhance macrophage bactericidal capacity	[116]..................
IL-4	Th2 cells	Motivate macrophage endocytosis function	[118]..................
IL-10	Treg cells, macrophages	Reduce Th2 and Th17 cells, suppress macrophage proliferation	[110, 125]
IL-12	Macrophages	Enhance type 1 inflammation	[99]..................
IL-13	Th2 cells	Enhance M2 macrophages	[119]..................
IL-17	Th17 cells, macrophages	Mediate neutrophilic inflammation and increase macrophages	[108, 122]
IL-18	Macrophages	Drive type 2 inflammation and Th2 cell differentiation	[103]..................
IL-27	Macrophages	Suppress Th2-type inflammation	[113]..................
IL-33	Macrophages	Drive Th2-type inflammation	[104]..................
MIF	Macrophages	Activate CD4^+^T cells and Th2-associated cytokines	[96]..................
GM-CSF	Th cells	Initiate macrophage activation and secretion	[115]..................
TNF-*α*	Th1 cells, macrophages	Enhance macrophage activation and recruitment, alleviate Th1 and Th2-associated cytokines, IgE and airway remodeling	[107, 116, 117]
IFN-*γ*	Th1 cells	Enhance macrophage endocytosis function	[116]..................
TGF-*β*	Treg cells, macrophages	Suppress macrophages and develop Treg cell proliferation	[112, 125]
TSLP	Macrophages	Stimulate Th2 cell differentiation	[102]..................
Prostaglandin	Macrophages	Act on T cells in a paracrine manner	[97]..................
LTC_4_	Macrophages	Modulate Th2 cells and Th2-type inflammation	[105]..................
CXCR3L	Macrophages	Gather Th1 cells	[98]..................
MMP-2	Macrophages	Downregulate Th2-type inflammation	[106]..................
Tim	T cells	Regulate macrophage vitality	[114]..................
PPAR*γ*	Th2 cells	Increase M2 macrophages and AMs	[120]..................

## Data Availability

The data used to support the findings of this study are included within the article.
